# Enhancement of Palmarumycin C_12 _and C_13_ Production in Liquid Culture of the Endophytic Fungus *Berkleasmium* sp. Dzf12 by Oligosaccharides from Its Host Plant *Dioscorea zingiberensis*

**DOI:** 10.3390/molecules17043761

**Published:** 2012-03-26

**Authors:** Yan Li, Tijiang Shan, Yan Mou, Peiqin Li, Jianglin Zhao, Wensheng Zhao, Youliang Peng, Ligang Zhou, Chunbang Ding

**Affiliations:** 1 College of Biology and Science, Sichuan Agricultural University, Yaan 625014, China; 2 College of Agronomy and Biotechnology, China Agricultural University, Beijing 100193, China

**Keywords:** endophytic fungus, *Berkleasmium* sp. Dzf12, spirobisnaphthalene, palmarumycin C_12_, palmarumycin C_13_, crude oligosaccharide, polysaccharide, elicitation, *Dioscorea zingiberensis*

## Abstract

Three crude oligosaccharides were respectively prepared by acid hydrolysis of three polysaccharides, which were water-extracted polysaccharide (WEP), sodium hydroxide-extracted polysaccharide (SEP) and acid-extracted polysaccharide (AEP) from the rhizomes of *Dioscorea zingiberensis*. Among the three oligosaccharides, the crude oligosaccharide prepared by acid hydrolysis of WEP was found to be the most efficient elicitor to enhance the production of palmarumycins C_12_ and C_13_ in liquid culture of endophytic fungus *Berkleasmium *sp. Dzf12. When OW was applied to the medium at 300 mg/L on day 3 of culture, the maximal yields of palmarumycin C_12_ (87.96 mg/L) and palmarumycin C_13_ (422.28 mg/L) were achieved on day 15 of culture, which were 9.83 and 3.24-fold in comparison with those (8.95 and 130.43 mg/L) of control, respectively. The results indicate that addition of the oligosaccharides from the host plant *D. zingiberensis* should be an effective strategy for enhancing production of palmarumycins C_12_ and C_13_ in liquid culture of endophytic fungus *Berkleasmium *sp. Dzf12.

## Abbreviations

OW: crude oligosaccharide prepared by acid hydrolysis of the water-extracted polysaccharide
OS: crude oligosaccharide prepared by acid hydrolysis of the sodium hydroxide-extracted polysaccharide
OA: crude oligosaccharide prepared by acid hydrolysis of the acid-extracted polysaccharide
WEP: water-extracted polysaccharide
SEP: sodium hydroxide-extracted polysaccharide
AEP: acid-extracted polysaccharide
TFA: trifluoroacetic acid

## 1. Introduction

Plant endophytic fungi, which inhabit normal tissues of host plants without causing apparent symptoms of pathogenesis [1], are novel and rich sources of bioactive natural products [2,3,4,5]. In connection with our ongoing search for bioactive metabolites from an endophytic fungus *Berkleasmium *sp. Dzf12, isolated from the healthy rhizomes of the medicinal plant *Dioscorea zingiberensis*, five spirobisnaphthalenes have been successfully obtained that show antimicrobial activity [6]. Spirobisnaphthalenes are a group of fungal secondary metabolites, consisting of 1,8-dihydroxynaphthalene-derived spiroketal units linked to a second oxidized naphthalene moiety, that show a great variety of biological activities such as antitumor, antibacterial, antifungal, antileishmanial, enzyme-inhibitory, and other properties that suggest potential applications in agriculture, medicine and the food industry [7,8]. Among the spirobisnaphthalenes isolated from *Berkleasmium *sp. Dzf12, both palmarumycins C_12_ and C_13_ were found to be the predominant components. Besides our previous study [6], palmarumycin C_12_ has also been isolated from the fungi *Coniothyrium *sp. [9] and *Sphaeropsidales* sp. (F-24'707, *Cladosporium chlorocephalum*) [10]. Palmarumycin C_12_ showed antifungal activity on *Ustilago violacea* and *Eurotium repens* [9]. Palmarumycin C_13_ (also variously named Sch 53514, diepoxin ζ and cladospirone bisepoxide) has also been isolated from other fungi such as *Coniothyrium *sp. [9], a non-sporulating fungus LL-07F275 [11], *Nattrassia mangiferae* [12], *Cladosporium chlorocephalum* (F-24'707) [10,13,14,15]. Palmarumycin C_13_ exhibited obvious antibacterial and antifungal [6,11], anti-tumor activity and inhibitory activity on phospholipase D (PLD) [12].

In order to speed up application of palmarumycins C_12_ and C_13_, one of the most important approaches is to increase yields of palmarumycins C_12 _and C_13_ in fermentation culture of *Berkleasmium* sp. Dzf12. Many strategies have been developed to increase metabolite yield in microorganism or plant cultures, which include optimization of medium, utilization of two-phase culture systems, addition of precursors, as well as application of elicitation [16,17]. Among these, elicitation has been regarded as a convenient and effective approach [18,19,20]. The use of carbohydrates (*i.e.*, polysaccharides and oligosaccharides) for enhancing the production of secondary metabolites in plant or fungal cultures is promising and could have potential applications [21,22,23]. In fungal cultures, addition of the oligosaccharides prepared from sodium alginate by partial acid hydrolysis to *Penicillium chrysogenum *cultures could increase the production of penicillin G and its biosynthetic intermediates [24]. In addition, changes in microbial cell morphology, sporulation, production of pigments and transcriptional activity of the penicillin biosynthesis genes (*i.e.*, *pcbAB*, *pcbC*, and *penDE*) have also been reported in response to the addition of oligosaccharide elicitors [25,26]. In our previous study, an obvious enhancement of palmarumycin C_13_ accumulation was elicited in liquid culture of *Berkleasmium* sp. Dzf12 by water-extracted polysaccharide (WEP) at 400 mg/L, sodium hydroxide-extracted polysaccharide (SEP) at 100 mg/L, and acid-extracted polysaccharide (AEP) at 400 mg/L, which were prepared from the rhizomes of the host plant *D. zingiberensis* [27]. In this work, three crude oligosaccharides, namely OW, OS and OA, were respectively prepared by acid hydrolysis of three polysaccharides from the rhizomes of *D. zingiberensis*, which were WEP, SEP and AEP. The enhancing effects of the oligosaccharides on secondary metabolite production of *Berkleasmium* sp. Dzf12 were compared. To the best of our knowledge, there are no previous reports on the effect of the oligosaccharides as elicitors from the host plant on the accumulation of secondary metabolites in its endophytic fungus. The purpose of this study was to investigate the effects of oligosaccharides on palmarumycins C_12_ and C_13_ production in liquid culture of the endophytic fungus *Berkleasmium* sp. Dzf12. Secondly, it was to investigate whether these oligosaccharides have better stimulating effect on palmarumycin production than the precursor polysaccharides.

## 2. Results and Discussion

### 2.1. Effects of Oligosaccharides OW, OS and OA on Mycelia Growth and Palmarumycin Production

The optimal concentrations of oligosaccharide elicitors were determined based on their effects on palmarumycin production. The addition time of oligosaccharides was determined on day 3 of culture according to the growth curves of *Berkeasmium* sp. Dzf12 in the liquid culture [21].

**Table 1:** Effects of the oligosaccharides OW, OS and OA on mycelia growth and palmarumycins C_12_ and C_13_ production in liquid culture of *Berkleasmium* sp. Dzf12.

Oligo. Conc. (mg/L)		Mycelia biomass (g dw/L)	C_12_ content in mycelia (mg/g dw)	C_13_ content in mycelia (mg/g dw)	C_13_ yield in broth (mg/L)	C_12_ plus C_13_ yield (mg/L)
CK	0	6.84 ± 0.26 e	1.31 ± 0.08 h	3.24 ± 0.18 f	108.28 ± 6.00 e	139.45 ± 5.20 g
OW	200	11.48 ± 0.26 b	5.40 ± 0.24 c	8.21 ± 0.21 b	229.06 ± 6.62 b	385.28 ± 8.32 b
	400	13.90 ± 0.86 a	6.20 ± 0.31 b	9.92 ± 0.18 a	245.40 ± 4.69 a	469.33 ± 9.43 a
	600	14.06 ± 0.25 a	4.95 ± 0.18 d	6.50 ± 0.32 c	193.78 ± 10.41c	369.53 ± 16.80 b
OS	100	8.69 ± 0.26 d	2.42 ± 0.14 g	4.21 ± 0.14 e	169.23 ± 7.01 d	226.90 ± 8.35 f
	200	9.24 ± 0.29 cd	5.09 ± 0.19 cd	6.88 ± 0.06 c	232.16 ± 9.47 ab	340.96 ± 14.79 c
	400	9.55 ± 0.47 c	8.06 ± 0.30 a	5.22 ± 0.06 d	179.05 ± 7.21 d	305.83 ± 8.69 d
OA	200	8.80 ± 0.24 d	2.63 ± 0.10 fg	4.33 ± 0.16 e	176.69 ± 8.76 d	237.89 ± 9.33 f
	400	9.32 ± 0.37 cd	3.44 ± 0.17 e	6.29 ± 0.35 c	241.72 ± 7.16 ab	332.28 ± 5.92 c
	600	9.82 ± 0.30 c	2.93 ± 0.15 f	5.15 ± 0.12 d	205.61 ± 9.00 c	283.56 ± 10.75 e

Note: CK, a control without addition of oligosaccharide; Oligo., oligosaccharide; C_12_, palmarumycin C_12_; C_13_, palmarumycin C_13_; The values are expressed as means ± standard deviations (*n* = 3). Different letters indicate significant differences among the treatments in each column including different oligosaccharides and their concentrations at *p* = 0.05 level.

The increase in palmarumycin production is due to not only the mycelia growth but also palmarumycin content of the fungus. The effects of three oligosaccharides (*i.e.*, OW, OS and OA) on mycelia growth and palmarumycin production in *Berkeasmium* sp. Dzf12 liquid culture are presented in Table 1. All three oligosaccharides showed improving effects on mycelia growth and palmarumycin accumulation. The optimal concentrations to stimulate mycelia growth for OW, OS and OA were respectively at 600, 400 and 600 mg/L. However, the optimal concentrations to obviously increase total palmarumycin yield (palmarumycins C_12_ plus C_13_) for OW, OS and OA were respectively at 400, 200 and 400 mg/L.

**Figure 1:**
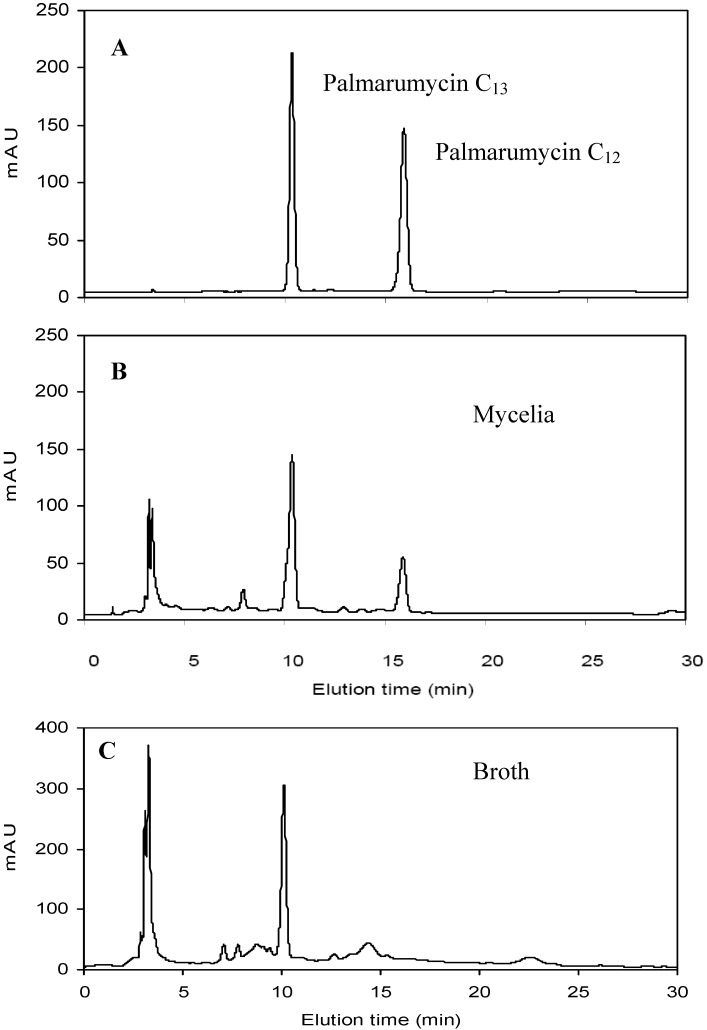
HPLC profiles of the extracts of mycelia and broth for palmarumycins C_12_ and C_13_ analysis: palmarumycins C_12_ and C_13_ (**A**), mycelia extract from batch culture on day 13 (**B**), broth extract from batch culture on day 13 (**C**). Elution time (retention time) for palmarumycins C_12_ and C_13_ were 15.911 min and 10.326 min, respectively. Palmarumycin C_12_ was undetectable in the broth extract.

Among the three oligosaccharides, OW was the most effective elicitor to enhance mycelia growth and palmarumycin production. Feeding with 400 mg/L of OW on day 3 increased intracellular palmarumycin C_12_ yield by 8.60-fold (86.15 mg/L *versus* 8.97 mg/L), and total palmarumycin C_13_ yield (intracellular palmarumycin C_13_ in mycelia plus extracellular palmarumycin C_13_ in medium) by 1.94-fold (383.18 mg/L *versus* 130.48 mg/L). Correspondingly, total palmarumycin (palmarumycins C_12_ plus C_13_) yield of endophyte Dzf12 liquid cultures was increased to reach 469.33 mg/L, which was 3.37-fold of control yield (139.45 mg/L). Very interestingly, palmarumycin C_12_ was undetectable in the broth extract which means it was not secreted from the intracellular to the extracellular. The HPLC profiles of the extracts of mycelia and broth along with palmarumycins C_12_ and C_13_ standards are shown in Figure 1.

### 2.2. Effects of Oligosaccharide OW Addition Time on Mycelia Growth and Palmarumycin Production

Addition time of the elicitor has been considered as a main factor affecting its elicitation effect on the biosynthesis of secondary metabolites [28,29]. The oligosaccharide OW obtained by acid hydrolysis of WEP was the most effective elicitor to improve palmarumycin biosynthesis. Hence, addition time of OW and its concentration were further optimized. As the 3-day-old cultures treated with 600 mg/L of OW could diminish palmarumycin production (Table 1), the highest concentration of OW in subsequent studies was limited at 500 mg/L.

**Figure 2:**
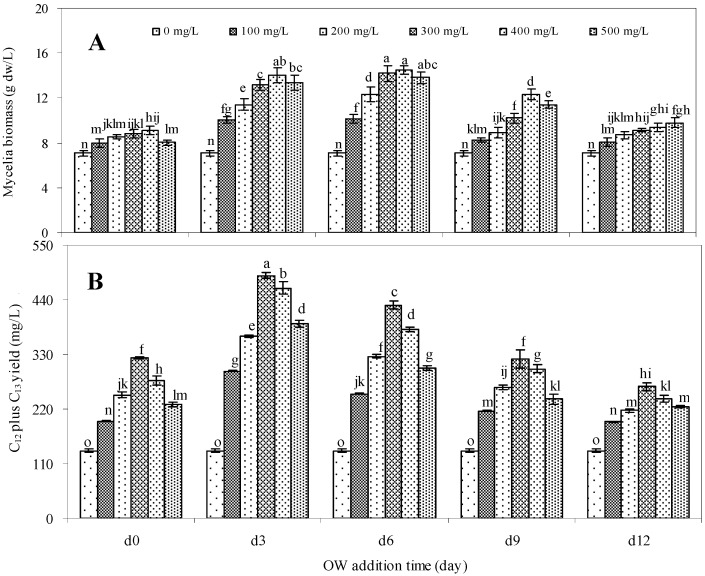
Effects of different concentrations of OW and its addition time on mycelia growth (**A**) and palmarumycins C_12_ plus C_13_ yield (**B**) in liquid culture of *Berkleasmium* sp. Dzf12. All cultures were harvested on day 13. The error bars represent standard deviations (*n* = 3). Different letters indicate significant differences among the treatments at *p* = 0.05 level.

Figure 2 shows the effects of OW on mycelia growth, palmarumycin content in mycelia, and palmarumycin yield in *Berkleasmium* sp. Dzf12 liquid cultures, which were dependent on both OW concentrations (100, 200, 300, 400 and 500 mg/L) and its addition time (added on day 0, 3, 6, 9 and 12). As shown in Figure 2A, when the cultures were fed with 400 mg/L of OW on day 6, the mycelia biomass was 2.05-fold of control (14.49 g dw/L *versus* 7.08 g dw/L). The palmarumycins C_12_ and C_13_ production was also effectively enhanced by OW. With 300 mg/L of OW fed on day 3, the highest contents of palmarumycins C_12_ (10.30 mg/g dw) and C_13 _(6.33 mg/g dw) in mycelia were obtained, which were respectively 3.12 and 4.83-fold in comparison with those produced in the control (3.40 and 1.31 mg/g dw). The total palmarumycin yield (palmarumycins C_12_ plus C_13_) was improved to reach 488.48 mg/L, which was about 3.60-fold of control yield (135.57 mg/L) (Figure 2B).

### 2.3. Kinetics of Mycelia Growth and Palmarumycin Accumulation after Treatment with Oligosaccharide OW

**Figure 3:**
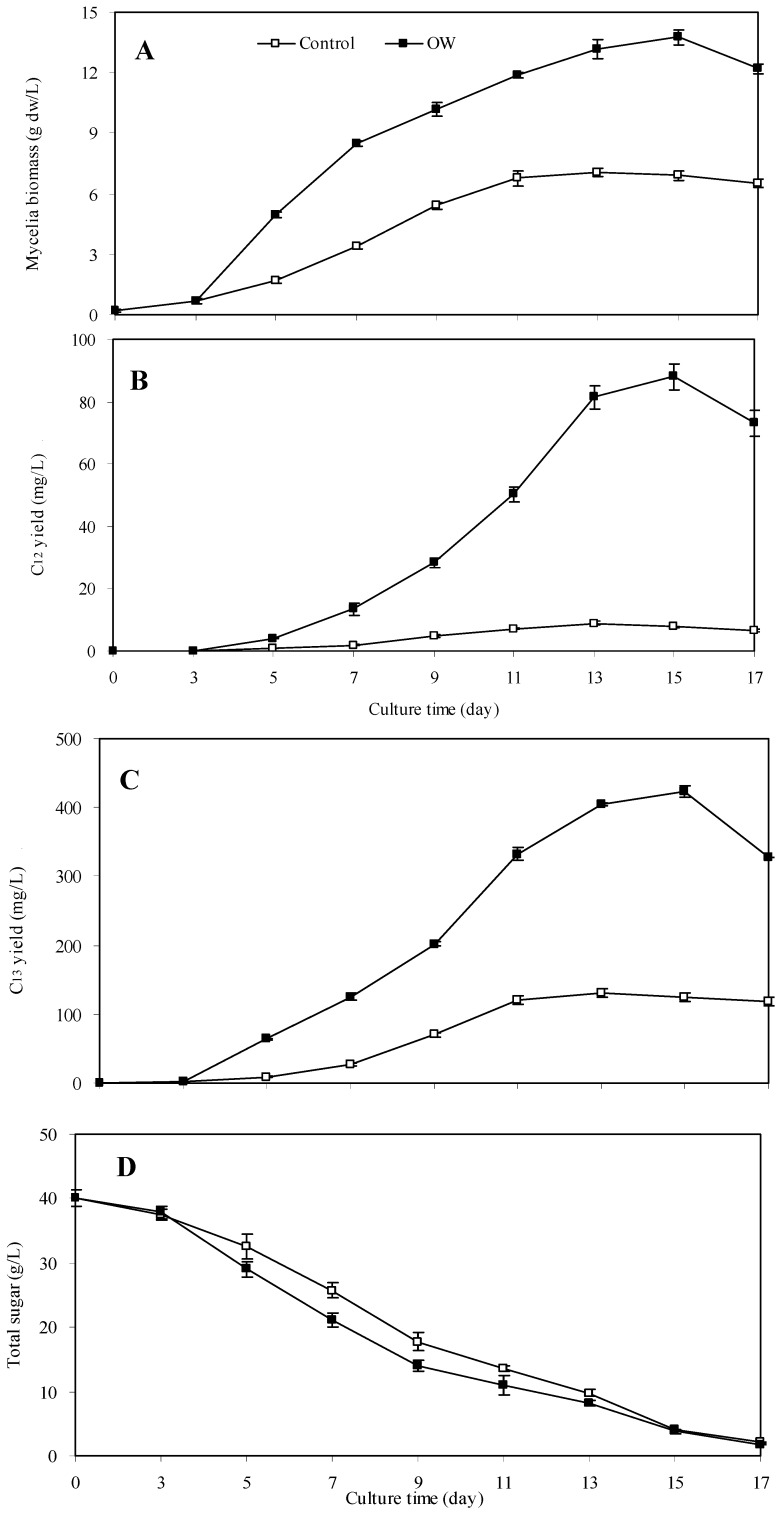
Kinetics of mycelia growth (**A**), palmarumycin C_12_ accumulation (**B**), palmarumycin C_13_ accumulation (**C**), and total sugar content (**D**) after treatment with OW at 300 mg/L on day 3 in liquid culture of *Berkleasmium* sp. Dzf12. The period of culture was in 17 days. The error bars represent standard deviations (*n* = 3).

The maximal palmarumycin accumulation was obtained when the cultures were treated with OW at 300 mg/L on day 3 (Figure 2). So the kinetic studies of mycelia growth, palmarumycin accumulation and glucose consumption in liquid culture of *Berkleasmium* sp. Dzf12 elicited by OW at 300 mg/L added on day 3 were investigated, which are shown in Figure 3.

The maximal mycelia biomass (13.76 g dw/L) was obtained on day 15, about 1.95-fold of control (7.04 g dw/L) (Figure 3A On day 15 of culture, palmarumycin C_12_ yield reached the maximum (87.96 mg/L), and palmarumycin C_13_ yield also reached the highest value (422.28 mg/L) (Figure 3B and C). The total palmarumycin yield was increased to the maximum on day 15 of culture, which was 510.24 mg/L, about 3.66-fold of control (139.38 mg/L). As shown in Figure 3D, the total sugar was consumed at a slightly higher rate with OW elicitation on day 5, and the trend maintained till day 11 in comparison with the control. The mycelia growth against total sugar consumption was 0.23 g dw/g for the control and 0.37 g dw/g for the cultures treated with OW, while the corresponding total palmarumycin yield on total sugar consumption was 4.61 mg/g (for the control) and 14.09 mg/g (for the cultures treated with OW). It indicated that the increase in both mycelia growth and palmarumycin yield was due to the elicitation of OW added in trace amounts (mg/L) but not as carbon source [24,30].

## 3. Experimental

### 3.1. Endophytic Fungus and Culture Conditions

The endophytic fungus *Berkleasmium* sp. Dzf12 (GenBank accession number EU543255) was isolated from the healthy rhizomes of the medicinal plant *Dioscorea zingiberensis* C. H. Wright (Dioscoreaceae) in our previous study [6,31]. The living culture has been deposited in China General Microbiological Culture Collection Center (CGMCC) under the number of CGMCC 2476. It was also maintained on potato dextrose agar (PDA) slants at 25 °C. For preparation of the inoculum, 4 disks (about 5 mm) of the mycelia of Dzf12 were transferred into each Erlenmeyer flask containing 100 mL of potato dextrose broth. The flasks were then placed on a rotary shaker at 150 rpm and 25 °C for 4 days. All the experiments were carried out in Erlenmeyer flasks (150 mL) containing 30 mL of fermentation medium, where liquid medium composition was (in g/L): glucose 40, peptone 10, KH_2_PO_4_ 1.0, MgSO_4_·7H_2_O 0.5, FeSO_4_·7H_2_O 0.05, pH 6.5. Three percent (v/v) of seed suspension cultures was inoculated. The Erlenmeyer flasks were incubated in darkness on a rotary shaker at 150 rpm and 25 °C.

### 3.2. Preparation of Oligosaccharides

Three crude oligosaccharides, OW (6.20 g), OS (5.25 g), OA (5.10 g) were respectively obtained by acid hydrolysis of 15 g of three freeze-dried polysaccharides, which were water-extracted polysaccharide (WEP), sodium hydroxide-extracted polysaccharide (SEP) and acid-extracted polysaccharide (AEP). These polysaccharides were prepared from the rhizomes of *D. zingiberensis* as described previously [27]. The oligosaccharides were prepared according to the methods of Zhang *et al.* [19] and Li *et al.* [29]. Each polysaccharide (WEP, SEP and AEP) was placed into a glass bottle treated with 2.17 mol/L trifluoroacetic acid (TFA) at 90 °C for 3 h in a water bath. The bottles were shaken every half hour and placed on an ice bath at the end of hydrolysis. The acid was removed under reduced pressure by repeated evaporation with MeOH. Three volumes of 95% EtOH were added to the hydrolysates to precipitate the non-hydrolyzed polysaccharides. The supernatant was concentrated. The values of anthrone-positive carbohydrate were 99.56%, 98.84% and 99.01% for the crude oligosaccharides from WEP, SEP and AEP, respectively. The obtained oligosaccharides were subjected to thin-layer chromatography (TLC) detection with *n*-butanol-ethyl acetate-acetic acid-water (3.0:0.5:1.7:4.1, v/v) as the developing agent. The reference oligosaccharides were kindly supplied by Prof. Shilin Wang of Kunming Institute of Botany, Chinese Academy of Sciences. The degrees of polymerization (DPs) of the oligosaccharides were at ranges of 2 to 12. The oligosaccharides were stored as solutions at -20°C, and sterilized by filtration before use.

### 3.3. Application of Oligosaccharides

Stock solutions (100 mg of carbohydrate equivalent per milliliter) were prepared by dissolving each crude oligosaccharide (*i.e.*, OW, OS and OA) in distilled water, and the pH was adjusted to 6.5. The solutions were sterilized by filtrating through a microfilter (pore size, 0.45 µm), diluted with sterile water into different concentrations, and then stored at 4 °C before use. Based on our previous research (data not shown), the oligosaccharide solutions were added to 3-day-old cultures with the final concentrations of 200, 400 and 600 mg/L for OW or OA, and 100, 200 and 400 mg/L for OS, respectively. As OW was found to be the most effective elicitor (data shown in Table 1), OW was applied in the next experiments at five concentrations (100, 200, 300, 400 and 500 mg/L) on days 0, 3, 6, 9 and 12 of culture, respectively. Furthermore, the kinetics of the mycelia growth and palmarumycin accumulation was investigated when OW was added on day 3 of culture at a concentration of 300 mg/L. Each flask was worked up separately.

### 3.4. Determination of Mycelia Biomass

The mycelia of *Berkleasmium* sp. Dzf12 were separated from the liquid medium by filtration under vacuum and rinsed thoroughly with distilled water, and then dried at 50–55 °C in an oven to a constant dry weight (dw).

### 3.5. Extraction and Quantification of Palmarumycins C_12_ and C_13_

Palmarumycin extraction and determination was carried out as previously described [21,27,32]. Briefly, 50 mg of dry mycelia powder was added into a tube with 5 mL of methanol-chloroform (9:1, v/v), and then subjected to ultrasonic treatment (three times, 60 min each). After removal of the solid by filtration, the filtrate was evaporated to dryness and re-dissolved in 1 mL of methanol. For quantitative analysis of palmarumycins C_12 _and C_13_ in broth, 5 mL of the culture broth without mycelia was evaporated to dryness and extracted with 5 mL of methanol-chloroform (9:1, v/v) in an ultrasonic bath (three times, 60 min each), and the liquid extract was then evaporated to dryness and re-dissolved in 1 mL of methanol.

Palmarumycin content was analyzed by the high performance liquid chromatography (HPLC) system (Shimadzu, Japan), which consisted of two LC-20AT solvent delivery units, an SIL-20A autosampler, an SPD-M20A photodiode array detector, and CBM-20Alite system controller. The reversed-phase Agilent TC-C_18_ column (250 mm × 4.6 mm i.d., particle size 5 μm) was used for separation by using a mobile phase of methanol-H_2_O (50:50, v/v) at a flow rate of 1 mL/min. The temperature was maintained at 40 °C, and UV detection at 226 nm. The sample injection volume was 10 μL. The LCsolution multi-PDA workstation was employed to acquire and process chromatographic data. Palmarumycins C_12_ and C_13 _were detected and quantified with the standards prepared according to the method of Cai *et al.* [6].

### 3.6. Physicochemical and Spectroscopic Data of Palmarumycins C_12_ and C_13_

**Figure 4:**
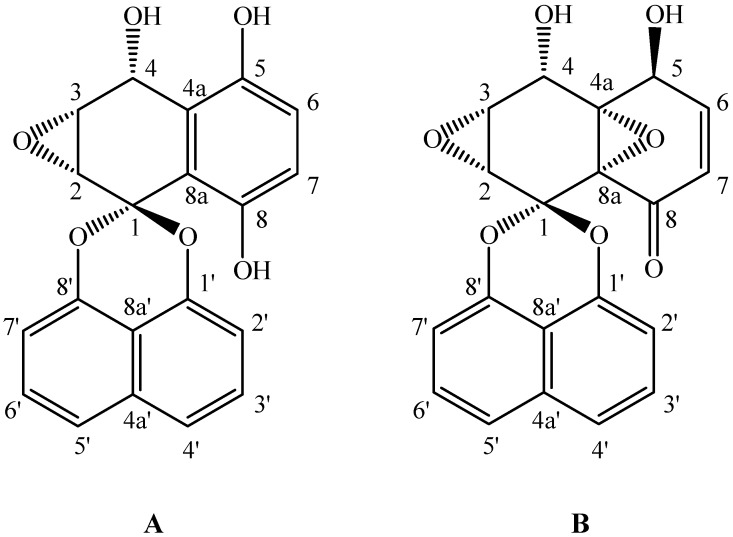
Chemical structures of palmarumycins C_12_ (**A**) and C_13_ (**B**).

Palmarumycin C_12_ was obtained as a white amorphous powder (MeOH), and mp 199-201 °C. Its molecular formula was determined as C_20_H_14_O_6_ (*m/z* 373.06805 [M+Na]^+^, calcd. 373.06826) by its HR-ESI-MS. ^1^H-NMR (DMSO-*d*_6_, 400 MHz) *δ* (ppm): 9.21 (1H, br. s, 5-OH), 8.81 (1H, s, 8-OH), 7.59 (lH, d, *J* = 8.2 Hz, 4'-H), 7.57 (lH, d, *J *= 8.2 Hz, 5'-H), 7.51 [1H, pseudo-t (dd), *J* = 7.4, 8.5 Hz, 3'-H], 7.49 [1H, pseudo-t (dd), *J* = 7.4, 8.5 Hz, 6'-H], 7.01 (1H, d, *J *= 7.4 Hz, 2'-H), 6.96 (1 H, d, *J* = 7.4 Hz, 7'-H), 6.81 (1H, d, *J* = 8.7 Hz, 7-H), 6.76 (1H, d, *J *= 8.7 Hz, 6-H), 6.46 (1H, br. s, 4-OH), 5.31 (l H, d, *J* = 2.6 Hz, 4-H), 3.64 (1H, d, *J* = 4.3 Hz, 2-H), 3.61 (1H, dd, *J* = 2.9, 4.3 Hz, 3-H). ^13^C-NMR (DMSO-*d*_6_, 100 MHz) *δ* (ppm): 97.2 (C-1), 52.4 (C-2), 54.0 (C-3), 64.4 (C-4), 121.5 (C-4a), 148.6 (C-5), 117.4 (C-6), 117.4 (C-7), 150.0 (C-8), 118.3 (C-8a), 147.4 (C-1'), 108.8 (C-2'), 127.9 (C-3'), 120.3 (C-4'), 133.9 (C-4a'), 120.1 (C-5'), 127.7 (C-6'), 108.6 (C-7'), 147.4 (C-8'), 112.1 (C-8a'). The structure (shown in Figure 4) of palmarumycin C_12_ was confirmed by comparison with literature data [9,33].

Palmarumycin C_13_ was obtained as colorless crystals (MeOH), mp 230 °C. Its molecular formula was determined as C_20_H_14_O_7_ (*m/z* 405.03720 [M+K]^+^, calcd. 405.03711) by its HR-ESI-MS. ^1^H-NMR (DMSO-*d*_6_, 400 MHz) *δ* (ppm): 7.64 (lH, d, *J* = 7.4 Hz, 4'-H), 7.62 (lH, d, *J* =7.4 Hz, 5'-H), 7.52 [2H, pseudo-t (dd), *J* = 7.4, 8.5 Hz, 3'-H, 6'-H], 7.10 (1H, d, *J *= 7.5 Hz, 2'-H), 7.04 (1H, d, *J* = 7.6 Hz, 7'-H), 6.74 (1H, dd, *J* = 4.9, 10.5 Hz, 6-H), 6.24 (1H, d, *J* = 7.8 Hz, 4-OH), 6.00 (1H, d, *J* = 7.5 Hz, 5-OH), 5.88 (1H, d, *J* = 2.2, 10.5 Hz, 7-H), 4.98 (lH, d, *J* = 7.6 Hz, 4-H), 4.69 (1H, m, 5-H), 3.17 (2H, d, *J* = 5.2 Hz, 2-H, 3-H). ^13^C-NMR (DMSO-*d*_6_, 100 MHz) *δ* (ppm):95.1 (C-1), 52.7 (C-2), 55.2 (C-3), 59.7 (C-4), 70.7 (C-4a), 60.6 (C-5), 125.3 (C-6), 144.8 (C-7), 188.8 (C-8), 62.2 (C-8a), 145.2 (C-l'), 109.1 (C-2'), 127.9 (C-3'), 120.7 (C-4'), 133.8 (C-4a'), 120.7 (C-5'), 127.7 (C-6'), 108.7 (C-7'), 145.5 (C-8'), 111.5 (C-8a'). The structure (shown in Figure 4) of palmarumycin C_13_ was confirmed by comparison with literature data [9,34].

### 3.7. Measurement of Total Sugar Content

Total sugar content in the medium was determined by anthrone test using glucose as the reference [18]. According to the method of Zhu *et al.* [35], mycelia growth against total sugar consumption was calculated as: (maximum mycelia biomass—Initial mycelia biomass)/(initial total sugar concentration—Residual total sugar concentration when the maximal mycelia biomass was obtained). Palmarumycin yield against total sugar consumption was calculated as: (maximum total palmarumycin yield—Initial total palmarumycin yield)/(initial total sugar concentration—Residual total sugar concentration when the maximal total palmarumycin yield was obtained).

### 3.8. Statistical Analysis

All experiments were carried out in triplicate, and the results were represented by their mean values and the standard deviations (SD). The data were submitted to analysis of variance (one-way ANOVA) to detect significant differences by PROC ANOVA of SAS version 8.2. The term significant has been used to denote the differences for which *p *≤ 0.05.

## 4. Conclusions

In this work, the oligosaccharides prepared from the polysaccharides isolated from the rhizomes of *D. zingiberensis* were successfully applied to the liquid cultures of *Berkleasmium* sp. Dzf12 for enhancing the production of palmarumycins C_12_ and C_13_. There were some reports about the oligosaccharide elicitation of fungal secondary metabolite production, such as the oligosaccharides derived from sodium alginate and locust-bean gum [24,25]. However, there are few reports about the effects of the oligosaccharides from plants on the secondary metabolism of their endophytic fungi. Oligosaccharides OW, OS and OA from *D. zingiberensis* had obvious enhancing effects on palmarumycin biosynthesis in *Berkleasmium* sp. Dzf12, although the elicitation mechanism was not clear. As the oligosaccharide employed in this study was a mixture, further separation and identification of the bioactive oligosaccharide monomers are necessary. The results indicate that enhancement of palmarumycin accumulation in liquid culture of *Berkleasmium* sp. Dzf12 by the oligosaccharides from host plant *D. zingiberensis* could be an effective strategy for large-scale production of palmarumycins C_12_ and C_13_ in the future. By comparing with our previous study on the enhancing effects of the polysaccharides from *D. zingiberensis* on palmarumycin C_13_ production [27], the oligosaccharide (*i.e.*, OW) has a better stimulating effect than its corresponding polysaccharide (*i.e.*, WEP). This indicated that the oligosaccharide fragments in polysaccharide may be the active components to stimulate secondary metabolism that should be further studied.
